# The complete chloroplast genome of *Clerodendrum japonicum* (Thunb.) Sweet, a traditional Chinese medicinal plant

**DOI:** 10.1080/23802359.2021.1885316

**Published:** 2021-03-15

**Authors:** Xiaofei Long, Yan Pan, Yuhao Weng, Zhaodong Hao, Daiquan Ye, Yunfei You, Jinhui Chen, Jisen Shi

**Affiliations:** a Key Laboratory of Forest Genetics & Biotechnology of Ministry of Education, Co-Innovation Center for Sustainable Forestry in Southern China, Nanjing Forestry University, Nanjing, China; b National Germplasm Bank of Chinese fir at Fujian Yangkou Forest Farm, Nanping, China

**Keywords:** Chloroplast genome, *Clerodendrum japonicum*

## Abstract

*Clerodendrum japonicum* (Thunb.) sweet, a member of Verbenaceae, is a traditional Chinese medicinal plant mainly distributed in tropical and subtropical Asia. Herein, we reported the complete chloroplast genome sequence of *C. japonicum*. The size of the chloroplast genome is 152,171 bp in length, including a large single-copy region (LSC) of 83,415 bp, a small single-copy region (SSC) of 17,318 bp, which was separated by a pair of inverted repeated regions of 25,719 bp. The *C. japonicum* chloroplast genome encodes 133 genes, including 88 protein-coding genes, 37 tRNA genes, and eight rRNA genes. The phylogenetic tree showed that *C. japonicum* is closely related to *C. mandarinorum* and *C. yunnanense.*

*Clerodendrum japonicum* (Thunb.) sweet is a shrub belonging to Verbenaceae family and is mainly distributed in South and East Asia. *Clerodendrum japonicum* is an excellent ornamental plant, and the whole plant can be used for medicine (Flora of China Editorial committee, Zhongguo Zhiwu Zhi). More than 30 compounds such as flavonoid compounds, phenylpropanoid glycosides, and terpenoids have been isolated and identified from *C. japonicum* (Zhang et al. 2018), which lays a foundation for elucidating its pesticide effect. At present, there are few information about the genomic information of *Clerodendrum*, including only two sequences of chloroplast genome, which were used as references in this study. Previous study showed the delimitations were more inclusive than others throughout *Clerodendrum* taxonomy, sequence data from the chloroplast *ndhF* gene and aligned *ITS* provided some shreds of evidence that *Clerodendrum* was polyphyletic (Steane et al. 2004). In our study, the complete chloroplast genome sequence of *C. japonicum* is constructed for better understanding the evolution of *C. japonicum* and its genus, and also providing significant information for the germplasm resource.

Fresh leaves of *C. japonicum* were collected from the Yangkou Forest Farm, in Fujian province, China, located at 117.30–l18.14E, 26.39–27.12N, and the voucher specimen deposited at Key Laboratory of Forest Genetics & Biotechnology of Ministry of Education, Nanjing Forestry University (Specimen code Cg_*CJ*). Total genomic DNA was extracted and then used for genome sequencing on a HiSeq Xten platform with the PE150 strategy, performed by Novogene (Nanjing), and finally, ∼5.34 GB of raw sequence data was achieved. ∼5.3 GB clean data was filtered with SAMtools (Aleman and Oufaska 2010) and Fastp (Chen et al. 2018). Then, software package velvet (Version. 1.2.10) (Zerbino and Birney 2008) was used to assemble the chloroplast genome *de novo*. The assembled genome was annotated using Geneious Prime (Version. 2020.2.4) and tRNA-SCAN (Chan and Lowe 2019), with the cp genome of *C. mandarinorum* (GenBank accession no.MN814861) and *C. yunnanense* (GenBank accession no. MN814862) as two references, and the annotation was corrected manually where necessary. The annotated complete cp genome of *C. japonicum* was submitted into GenBank with achieving the accession no. MW222242.

The complete circular cp genome of *C. japonicum* was 152,171 bp in length, containing a typical quadripartite structure, which is consists of two inverted repeat regions (IRs: 25,719 bp) separated by a large single-copy region (LSC: 83,415 bp) and a small single-copy region (SSC: 17,318 bp). The overall cp genome GC content is ∼38.1%, while the IR region has a higher GC content (∼43.3%) than the LSC region (∼36.1%) and the SSC region (∼31.9%). A total of 133 genes were successfully annotated containing 88 protein-coding genes, 37 tRNA genes, and 8 rRNA genes.

To better understand the phylogenetic position of *C. japonicum,* we generated *a* phylogenetic tree based on tandem sequences of 87 protein-coding genes without double-copy gene *rps12*, one of which existed as an incomplete copy and was identified as a trans-spliced gene. In this phylogenetic analysis, there are other 13 tandem sequences from Verbenaceae species, and one from *Capsicum annuum* belonging to Solanaceae species used as outgroup. All the 87 gene sequences were initially aligned using MUSCLE algorithm (Kumar et al. 2018) and then manually adjusted and combined using BioEdit (Version 7.2.5). A maximum likelihood (ML) tree was inferred using MEGA X (Kumar et al. 2018), with the combined rapid bootstrap (1000 replicates). Phylogenetic tree indicated that chloroplast sequences from the same genus can be clustered into one branch with high reliability, and *C. japonicum* was sister to *C. mandarinorum and C. yunnanense* (Figure 1). As an outgroup, *Capsicum annuum* cannot be clustered into Verbenaceae, and its gene has species-specific insertion or deletion: for example, insertions in *trnL–trnF* and *accD*, deletions in *petA–psbJ* and *ndhF–rpl32* (Jo et al. 2011).

**Figure 1.**
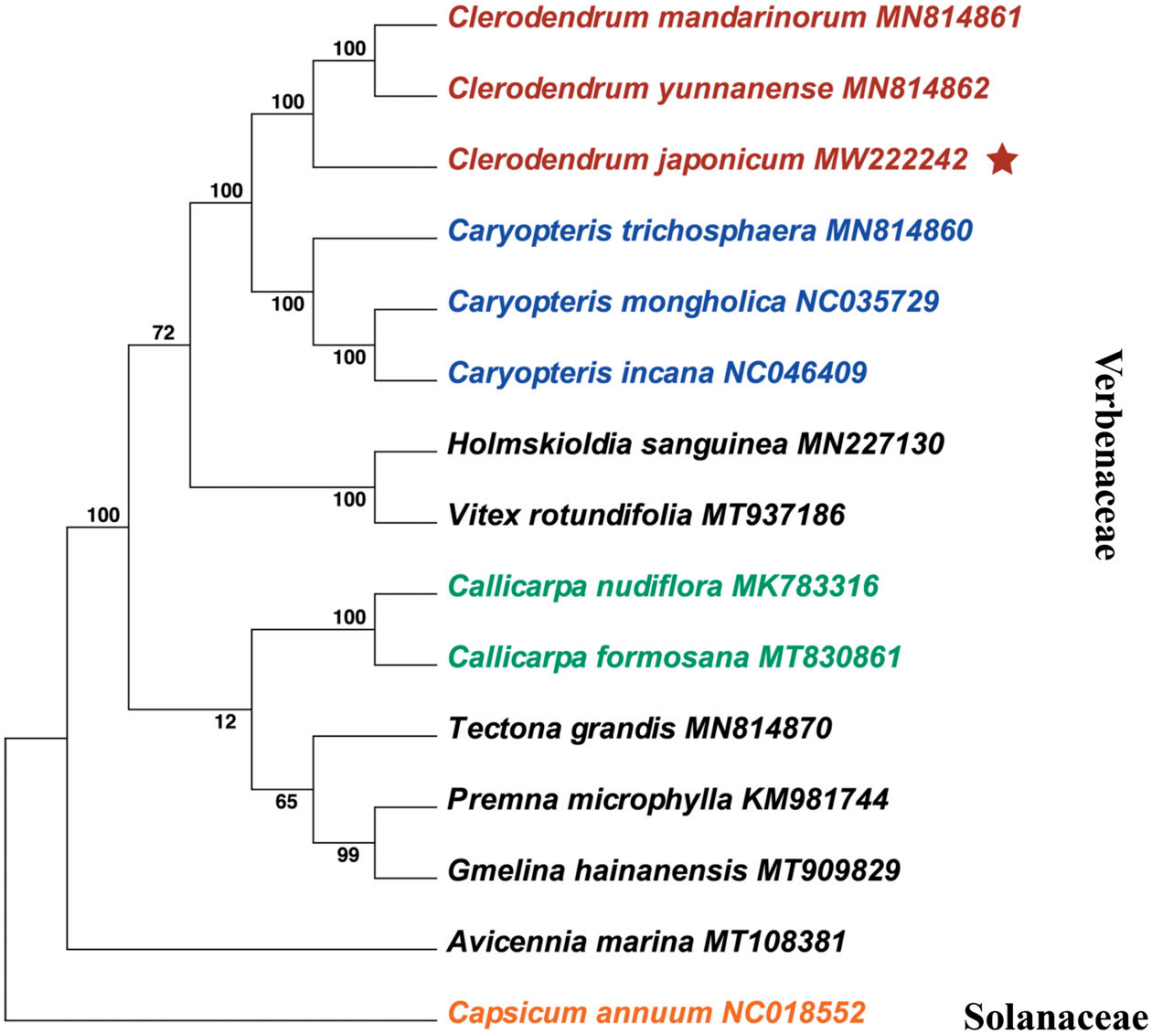
Maximum likelihood (ML) tree based on the tandem sequences combined with 87 protein-coding gene. The number along branches indicates bootstrap support value.

## Data Availability

The genome sequence data that support the findings of this study are openly available in GenBank of NCBI at (https://www.ncbi.nlm.nih.gov/) under the accession no.MW222242. The associated BioProject, SRA, and Bio-Sample numbers are PRJNA687386, SAMN17142233, and SRR13350637, respectively.
